# Best Practice in Systemic Therapy for Head and Neck Squamous Cell Carcinoma

**DOI:** 10.3389/fonc.2019.00815

**Published:** 2019-08-27

**Authors:** Sjoukje F. Oosting, Robert I. Haddad

**Affiliations:** ^1^Department of Medical Oncology, University Medical Center Groningen, University of Groningen, Groningen, Netherlands; ^2^Department of Medical Oncology, Dana-Farber Cancer Institute, Boston, MA, United States

**Keywords:** best practice, systemic treatment, chemotherapy, immunotherapy, head and neck cancer, squamous cell carcinoma

## Abstract

Treating head and neck cancer patients with systemic therapy is challenging because of tumor related, patient related and treatment related factors. In this review, we aim to summarize the current standard of care in the curative and palliative setting, and to describe best practice with regard to structural requirements, procedures, and monitoring outcome. Treatment advice for individual head and neck cancer patients is best discussed within a multidisciplinary team. Cisplatin is the drug of choice for concomitant chemoradiotherapy in the primary and postoperative setting, and also a main component of induction chemotherapy. However, acute and late toxicity is often significant. Checkpoint inhibitors have recently been proven to be active in the metastatic setting which has resulted in a shift of paradigm. Detailed knowledge, institution of preventive measures, early recognition, and prompt treatment of adverse events during systemic therapy is of paramount importance. Documentation of patient characteristics, tumor characteristics, treatment details, and clinical and patient reported outcome is essential for monitoring the quality of care. Participation in initiatives for accreditation and registries for benchmarking institutional results are powerful incentives for implementation of best practice procedures.

## Introduction

Patients with locally advanced or recurrent/metastatic head and neck squamous cell carcinoma (HNSCC) constitute a challenging population for systemic treatment because of tumor related, patient related and treatment related factors. The primary tumor can cause problems with eating, dysphagia and pain, resulting in significant weight loss already before diagnosis, while weight loss of more than 5% is an independent prognostic factor for worse progression free survival (1). Patients with advanced hypopharyngeal and laryngeal carcinomas can present with airway obstruction, or develop airway obstruction early during treatment and may require a tracheostomy. Patient related factors that can complicate systemic treatment are tobacco and alcohol addiction, co-morbidity and lack of a social network. In the curative setting, high-dose cisplatin concurrent with radiotherapy is the standard of care, either as primary treatment or after surgery. Chemoradiotherapy induces high rates of acute and late or long term adverse events. On the other hand, in the recurrent/metastatic setting, the field is rapidly evolving with the emergence of immune checkpoint inhibitors. Here, we summarize standard systemic treatment regimens, and describe best practice for administering systemic treatment with regards to structural requirements, procedures and monitoring outcome.

## Standard Systemic Treatment Regimens

### Locoregionally Advanced Disease

For patients with locoregionally advanced HNSCC with non-resectable tumors or in whom functional outcome of surgery is expected to be poor, primary concomitant chemoradiotherapy (CRT) with high-dose cisplatin (100 mg/m^2^) delivered every 3 weeks × 3 is the preferred treatment regimen (2–6). In Table 1, treatment regimens based on at least one phase III study are summarized. In oropharyngeal cancer patients accelerated fractionation radiotherapy over 6 weeks with two cycles of high-dose cisplatin resulted in similar outcome as conventional fractionation radiotherapy over 7 weeks with three cycles of high-dose cisplatin (10). Alternative concomitant systemic therapy regimens that improve overall survival compared to radiotherapy alone are carboplatin with infusional 5-fluorouracil (5FU) (13) or cetuximab (14). Based upon a lower level of evidence weekly cisplatin (40 mg/m^2^) (23–25), cisplatin with 5FU (26, 27), hydroxyurea with 5FU, cisplatin with paclitaxel (26, 27), or weekly carboplatin with paclitaxel can be considered (28). Benefit of concomitant chemotherapy decreases with age, and in a meta-analysis no benefit over locoregional treatment alone could be demonstrated for patients ≥70 years of age (29). Similarly, elderly patients do not benefit in the same way as younger patients from the addition of cetuximab to radiotherapy (14). This is paralleled by an increase in non-cancer related deaths in elderly patients. Proper selection of fit elderly patients with geriatric assessment might identify a subgroup that derives the same benefit as younger patients, but prospective data to support this is currently lacking. Treatment of elderly head and neck cancer patients has recently been extensively reviewed (30). Hypoxia modification with nimorazole during radiotherapy has been shown to improve locoregional control compared to radiotherapy alone (31) and is used in some countries as a standard of care. Patients with locoregionally advanced human papilloma virus (HPV) associated oropharyngeal cancer have significantly better outcome than patients with non-HPV related HNSCC, and treatment de-intensification strategies are under investigation. However, two randomized studies recently demonstrated that radiotherapy with cetuximab results in inferior overall survival compared to CRT with high-dose cisplatin, which therefore remains the standard of care (11, 12).

**Table 1:** Standard systemic treatment regimens for HNSCC^*^.

**Setting**	**Regimen (reference)**	**Dosing schedule**	**Remarks**
**Induction chemotherapy**			Benefit over CRT is unclear
	TPF (7)	Docetaxel 75 mg/m^2^ + cisplatin 100 mg/m^2^ on day 1 followed by continuous infusion of 5FU 1,000 mg/m^2^/day for 4 days every 3 weeks for three cycles	US regimen
	TPF (8, 9)	Docetaxel 75 mg/m^2^ + cisplatin 75 mg/m^2^ on day 1 followed by continuous infusion of 5FU 750 mg/m^2^ for 5 days, every 3 weeks for three cycles	European regimen
**Primary concomitant chemoradiotherapy/bioradiotherapy**			
	Cisplatin (4, 6, 10–12)	Cisplatin 100 mg/m^2^ on day 1, 22, and 43 during standard fractionated RT (or on day 1 and 22 during accelerated RT)	Preferred CRT regimen Accelerated RT plus 2 cycles cisplatin was not superior to standard fractionated RT plus three cycles cisplatin
	Carboplatin/5FU (13)	Carboplatin 70 mg/m^2^ on day 1–4, continuous infusion of 5FU 600 mg/m^2^/day on day 1–4 in week 1, 4, and 7 during RT	Has not been compared head to head with cisplatin
	Cetuximab (14)	Cetuximab 400 mg/m^2^ 1 week before start of RT and weekly 250 mg/m^2^ during RT	Inferior to cisplatin (for HPV related oropharyngeal cancer)
**Postoperative chemoradiotherapy**			
	Cisplatin (15–18)	Cisplatin 100 mg/m^2^ on day 1, 22, and 43 during RTCisplatin 50 mg flat dose weekly	Inferior LRC with weekly 30 mg/m^2^ cisplatin compared to 3-weekly 100 mg/m^2^ Superior LRC, OS and DFS with weekly 50 mg cisplatin compared to radiotherapy alone but has not been compared to 3-weekly 100 mg/m^2^
**Recurrent/metastatic palliative setting, 1st line**			
	Pembrolizumab (19)	Pembrolizumab 200 mg every 3 weeks	Approved by FDA but not (yet) by EMA Superior OS compared to EXTREME in patients with CPS ≥20 and in patients with CPS ≥1
	Platinum, 5FU and pembrolizumab (19)	Cisplatin 100 mg/m^2^ or carboplatin AUC 5 on day 1, plus 5FU 1,000 mg/m^2^/day on day 1–4, every 3 weeks for a maximum of six cycles plus pembrolizumab 200 mg every 3 weeks until progression	Approved by FDA but not (yet) by EMA Superior OS compared to EXTREME
	EXTREME (20)	Cisplatin 100 mg/m^2^ or carboplatin AUC 5 on day 1, plus 5FU 1,000 mg/m^2^/day on day 1–4, every 3 weeks for a maximum of six cycles plus cetuximab 400 mg/m^2^ at first dose, then 250 mg/m^2^ weekly until disease progression	
**Recurrent/metastatic palliative setting, 2nd line**			
	Nivolumab (21)	Nivolumab 3 mg/kg (can be replaced by 240 mg flat dose) every 2 weeks	After platinum containing chemotherapy
	Pembrolizumab (22)	Pembrolizumab 200 mg every 3 weeks	After platinum containing chemotherapy Europe: restricted to patients with PD-L1 TPS ≥ 50%

* *Based on at least one randomized phase III study. 5FU, 5-fluorouracil; AUC, area under the curve in mg per milliliter per minute; CPS, combined positive score for PD-L1 expression on tumor and immune cells; CRT, chemoradiotherapy; DFS, disease free survival; EMA, European Medicines Agency; FDA, US Food and Drug Administration; LRC, locoregional control; OS, overall survival; PD-L1, programmed death receptor ligand 1; RT, radiotherapy; TPF, docetaxel (Taxotere?), cisplatin (platinum), and 5FU; TPS, tumor proportion score (percentage of tumor cells with membranous PD-L1 staining)*.

Patients who undergo primary surgical treatment and have involved resection margins and/or extranodal extension of lymph node metastasis are at high risk of developing recurrent disease. Outcome in these patients is improved by the addition of concomitant high-dose cisplatin to postoperative radiotherapy (15, 16, 32). Results of studies with high-dose and low-dose concurrent cisplatin were recently summarized (33). Of the two randomized trials that have been reported, one study was not evaluable for efficacy due poor accrual (34). The second study compared 6–7 weekly cycles of 30 mg/m^2^ with three cycles of 100 mg/m^2^ cisplatin every 3 weeks (17). Locoregional control at 2 years was inferior in the low-dose arm (58.5 vs. 73.1%) and progression free survival and overall survival were numerically inferior but statistical significance was not reached for survival endpoints. It remains unclear to what extend the lower cumulative dose of the weekly regimen is responsible for inferior efficacy. Results of a third randomized phase II/III study comparing three times 100 mg/m^2^ with 7 weekly cycles of 40 mg/m^2^ cisplatin in the postoperative setting are awaited (35).

Induction chemotherapy followed by either radiotherapy alone, or radiotherapy with cetuximab, or CRT with weekly carboplatin, can be used as an organ preservation strategy. However, its benefit over CRT alone is not clear at this stage with conflicting phase III studies and heterogenous patient populations on these trials and the role of induction chemotherapy is therefore debated (36, 37). If induction chemotherapy is chosen, docetaxel with cisplatin and 5FU (TPF) is the preferred combination (7–9). In the United States (US) three cycles of docetaxel 75 mg/m^2^ plus cisplatin 100 mg/m^2^ followed by continuous infusion of 1,000 mg/m^2^ 5FU for 4 days every 3 weeks is used (7), while in Europe four 3-weekly cycles of docetaxel 75 mg/m^2^, cisplatin 75 mg/m^2^ followed by continuous infusion of 750 mg/m^2^ 5FU for 5 days is used (8).

### Recurrent/Metastatic Disease

For patients with metastatic HNSCC, or recurrent disease that is not amenable to curative intent treatment, the EXTREME regimen consisting of cisplatin or carboplatin with 5FU and cetuximab followed by cetuximab maintenance has been the standard first-line treatment for the last decade (20). Based upon a lower level of evidence, other chemotherapy combinations or single-agent treatment options can be considered (2). In patients who progress after platinum containing chemotherapy, treatment with an anti-programmed death 1 (PD1) antibody improves overall survival and induces durable responses in a subgroup of patients with a lower rate of grade 3–4 adverse events compared to investigator's choice systemic therapy (21, 22, 38, 39). Nivolumab was shown to improve overall survival irrespective of HPV status or programmed death ligand 1 (PD-L1) expression with better preservation of quality of life compared to the control arm (38, 40). Pembrolizumab also improved overall survival, in the entire cohort and in the subgroups of patients with PD-L1 positive tumors (22). This led to approval of pembrolizumab for patients with a PD-L1 tumor proportion score (percentage of tumor cells with membranous PD-L1 staining) ≥50% in 2018 by the European Medicines Agency (EMA), while the FDA granted accelerated approval irrespective of PD-L1 expression back in 2016, based on the results of the phase 1b study (41). However, treatment paradigm for the recurrent/metastatic setting will likely change again soon, since the final analysis of the KEYNOTE 048 study in the first-line recurrent/metastatic setting indicated that compared with the EXTREME regimen, pembrolizumab plus platinum and 5FU followed by pembrolizumab maintenance had superior overall survival in the PD-L1 combined positive score (CPS) ≥20, CPS ≥1, and total populations with comparable safety (19). Pembrolizumab alone had superior overall survival in the CPS ≥20 and ≥1 populations, with non-inferior overall survival in the total population, and favorable safety compared to EXTREME (19) and is already mentioned in the NCCN clinical practice guideline (2).

## Structural Requirements

The first requirement for effective delivery of systemic therapy to HNSCC patients is identification of patients in whom systemic treatment is indicated. The best way of doing this is discussing every newly diagnosed patient, every patient with recurrent disease and every patient who requires a change in treatment plan, during a multidisciplinary team (MDT) conference. An MDT approach is associated with improved tumor staging, better adherence to quality indicators, more concomitant CRT, shorter time between surgery and adjuvant therapy, higher completion rate of adjuvant treatment, and most important: improved disease specific and overall survival (42–46). According to the Dutch guidelines, a head and neck oncology center should at minimum have in the team three head and neck surgeons (at least one otolaryngeal surgeon and one oral and maxillofacial surgeon), two reconstructive surgeons, two head and neck radiation oncologists, and at least one head and neck medical oncologist, dermatologist, head and neck radiologist, pathologist, nuclear medicine physician, oncology nurse/case manager, dietician, physiotherapist, speech-language pathologist, dentist-maxillofacial prosthodontist, psychologist, and social worker (47). This list closely resembles the core team defined in the Canadian guidelines (48, 49). The minimum recommended volume for medical oncologists who care for head and neck cancer patients is 25 per year, although scientific evidence to support this number is lacking (48–50). In the Netherlands, the minimum required volume for immunotherapy in a hospital is 20 patients per year, but this may include different cancer types.

With regards to the healthcare facility, the optimal situation is to have the pharmacy, the infusion facility, the radiation center, the inpatient ward, specialists for treatment of immunotherapy side effects, an intensive care unit, and a 24/7 emergency department in one center. This enables quick communication between health care professionals and prompt admission to address adverse events, which helps to keep treatment breaks to a minimum.

Specific information about treatment schedules, potential side effects, instructions on when to contact the oncology nurse or medical oncologist along with contact details, is of importance for patients. This can be digital or on paper.

## Best Practice Procedures

After discussing a patient within the MDT, it is recommended to file a report in the patient's records which accessible for every team member and contains tumor characteristics including TNM stage, patient characteristics such as co-morbidity, medical history, tobacco and alcohol consumption, treatment intent (curative or palliative), and treatment plan (50). If the treatment advice deviates from the guidelines, it is preferable to specify the reason for it.

A longer waiting time between histopathological diagnosis and start of primary treatment is independently associated with worse overall survival in patients with HNSCC (51). The median tumor volume doubling time was shown to be 99 days in a Danish cohort, but in the half with the fastest growing tumors this was 30 days (52). Therefore, starting treatment as quickly as possible will improve patient outcome.

If systemic therapy is recommended by the MDT, the patient is referred to the medical oncologist who will carefully evaluate if systemic treatment is feasible through assessing the performance status, co-morbidity, previous medical history, organ function, and current medication. For elderly patients, geriatric screening and/or comprehensive geriatric assessment is recommended (53). Vulnerability according to the G8 was found to be independently associated with worse overall survival and persistent lower quality of life in HNSCC patients who received curative intent (chemo)radiotherapy (54).

### Chemotherapy

In general, Eastern Cooperative Oncology Group (ECOG) performance status worse than 2 (where 2 is defined as ambulatory and able of all self-care but unable to carry out any work activities; up and about for >50% of waking hours) is considered a contraindication for chemotherapy. Furthermore, blood cell counts, renal function, electrolytes and liver tests need to be adequate, and have to be assessed before each cycle.

Nutritional status is of particular importance in HNSCC patients. The tumor itself can cause problems with chewing, odynophagia, and dysphagia which can result in malnutrition. In addition, tooth extractions are performed in many patients before start of radiotherapy, further limiting the ability to eat normally. Also treatment side effects, especially of concomitant CRT, can cause swallowing problems. In the acute phase this is mainly related to mucositis, while dry mouth and sticky saliva are prominent long term side effects. In order to secure nutrition during CRT, prophylactic placement of a percutaneous endoscopic gastrostomy (PEG) tube can be considered. In a randomized study prophylactic PEG tube placement resulted in less malnourished patients, longer enteral feeding and better quality of life at 6 months after treatment without increased risk of long-term dysphagia compared with a control group treated according to clinical practice (55, 56). However, not all patients need enteral feeding, and selection of patients at high risk for malnutrition based on weight loss before start of treatment, age and radiotherapy dose to the constrictor muscles, can be used to select patients for prophylactic PEG tube placement (57). Nasogastric tube feeding appears to be an effective alternative to maintain body weight and the optimal method for enteral feeding of HNSCC patients has not yet been determined (58).

It is recommended to dose chemotherapy on actual body weight or, in the case of carboplatin, on actual stable creatinine clearance. In order to check and sign that the right drug is given to the right patient at the right dose at the right moment, the pharmacist and the nurses at the infusion facility need to be informed which treatment protocol applies, what is the treatment cycle number and day, the date, the height of the patient, actual weight, body surface area and/or creatinine clearance, and whether or not a dose reduction is applied. Including this information in the prescription, and filing prescriptions in the patient records facilitates personalized treatment modifications.

Nausea is a prominent side effect of chemotherapy and cisplatin belongs to the high-emetic-risk antineoplastic agents. A combination of four drugs consisting of a neurokinin 1 receptor antagonist, a serotonin receptor antagonist, dexamethasone, and olanzapine is recommended for cisplatin (59). Carboplatin belongs to the moderate-emetic-risk category requiring a three-drug antiemetic regimen, and docetaxel, 5FU and cetuximab have a low-emetic-risk, however combinations and multiday regimens should be treated per day for the drug with the highest emetic risk, and for 2 days after the last dose (59).

In addition to general chemotherapy side effects, cisplatin can cause renal toxicity, hearing loss, and neuropathy, and it can provoke cardiovascular events. Therefore, audiometric testing and an electrocardiogram is recommended before start of treatment, and thereafter if clinically indicated. Before every cycle, presence of neuropathy has to be assessed and creatinine clearance should be ≥60 ml/min. Adequate intravenous hydration from 2 to 12 h prior until at minimum 6 h after the administration of cisplatin is essential to protect renal function, and forced diuresis with mannitol or diuretics may be required (60). Allergic reactions to platinum compounds can occur. Therefore, it is important to have medication and a protocol for treatment of allergic reactions readily available at the infusion facility.

5FU is degraded into inactive metabolites by the enzyme dihydropyrimidine dehydrogenase (DPD). Variations in the gene encoding DPD result in reduced enzyme activity, increased 5FU exposure and severe mucositis and hematologic toxicity. Prospective genotyping and upfront 5FU dose reduction in patients who carry a variant allele predicting reduced metabolism can prevent potentially lethal toxicity also in patients who undergo chemoradiation with a relatively low 5FU dose (61, 62). In intermediate metabolizers, a dose reduction of 25–50% is recommended, while in poor metabolizers with complete DPD deficiency, it is recommended to avoid 5FU (63).

Docetaxel can induce fluid retention and hypersensitivity reactions characterized by generalized erythema and hypotension. In order to reduce the risk and severity of these side effects, patients can to be treated with dexamethasone for 3 days, starting the night before docetaxel administration (64). A study in Chinese patients with head and neck cancer receiving TPF showed that lower dexamethasone doses than the recommended six doses of 8 mg (twice daily) did not increase the risk of severe hypersensitivity reactions (65). The risk of alopecia from docetaxel can be reduced by scalp cooling (66). However, because of tumor localization close to the scalp, reduced efficacy as a result of cooling is a concern and therefore scalp cooling is not recommended in HNSCC patients. For the TPF regimen, antibiotic prophylaxis with ciproflacin 500 mg orally twice daily, from day 5 to 15 for prevention of neutropenic infections was administered in the pivotal trial (8). If patients develop neutropenic fever or neutropenic infection, addition of granulocyte colony stimulating factor (G-CSF) after the next cycles is recommended (67). In a retrospective analysis, primary prophylactic G-CSF did not reduce the incidence of febrile neutropenia in patients treated with TPF and ciprofloxacin or levofloxacin (68). Like for cisplatin, neuropathy is also a frequent side effect of docetaxel and assessment before each cycle is recommended.

### Cetuximab

Cetuximab can induce severe infusion-related reactions, including anaphylactic reactions even within minutes of the start of the first infusion. In the registration study, an antihistamine was administered as premedication, followed by a test dose of 20 mg cetuximab in 10 min followed by 30 min of observation (69). Four out of 211 patients discontinued cetuximab because of a hypersentitivity reaction after the test dose or the first dose. The compendium advises premedication with an antihistamine and a steroid, as well as close monitoring and prompt treatment of allergic reactions (70). A frequent adverse event of EGFR targeting drugs is an acneiform skin rash. Prophylactic treatment with an oral antibiotic such as doxycycline or minocycline can be used to reduce the severity of the rash, although not all trials showed consistent results, however it is recommended to instruct patients about sunlight protection (71). Another frequently occurring side effect is hypomagnesemia, especially in patients who receive ≥7 cetuximab infusions and concurrent cisplatin or carboplatin (72). Intravenous supplementation may be required and it may take several weeks or months to resolve.

### Immune Checkpoint Inhibitors

Nivolumab, pembrolizumab, and other immune checkpoint inhibitors can cause a wide spectrum of immune related adverse events. The most frequently affected organs are the skin, the gastrointestinal tract, the lungs, and endocrine organs including thyroid, pituitary, and adrenal glands. Less commonly the musculoskeletal tract, nervous system, kidneys, eyes, and heart and blood vessels are affected. Some of these side effects are potentially lethal. Prompt treatment usually results in complete resolution, although endocrinopathies may require lifelong hormonal substitution. The European Society of Medical Oncology (ESMO) and the American Society of Clinical Oncology (ASCO) developed guidelines for management of immunotherapy side effects (73, 74). For grade 3–4 toxicity, consultation of organ specialists such as a dermatologist, gastroenterologist, endocrinologist, pulmonologist etc. is required, which implies that a multidisciplinary team with expertise in treatment of immunotherapy side effects has to be available. In contrast to chemotherapy and cetuximab, immune checkpoint inhibitors may be continued at first progression provided that the patient has not deteriorated, although the incidence of pseudoprogression appears to be low in HNSCC (75).

## Outcome

If there is suspicion of recurrent disease in patients treated with curative intent, imaging and biopsy is required for confirmation. In the palliative setting, assessment of disease progression and treatment response according to the Response Evaluation Criteria In Solid Tumors (RECIST) (76) is preferred, also for patients treated outside studies. For evaluation of immunotherapy, a consensus guideline called iRECIST has been developed to capture response patterns such as pseudoprogression that differ from response patterns to cytotoxic agents (77). Universal criteria for evaluation facilitate benchmarking of institutional results against data from other centers and comparison with the literature. For the same reason it is important to record the date of death and whenever possible, the cause of death in the patient file.

Documentation of complications, unexpected toxicity and serious toxicity of systemic treatment can improve safety of the individual patient, and prevent further damage. It also allows listing for periodical discussion of incidence and potential causes within the MDT. When these discussions are followed by implementation of strategies to lower complication risk, future patients will be better protected. In order to be able to compare incidence of severe toxicity and complications with the literature and with other centers, use of the Common Terminology Criteria for Adverse Events is recommended (78).

Next to medical outcome parameters, patient reported outcome measures (PROMs) are increasingly used to get insight in the impact of treatment on disease symptoms, functional ability, and quality of life (79, 80).

## Quality Assessment

**Figure 1:**
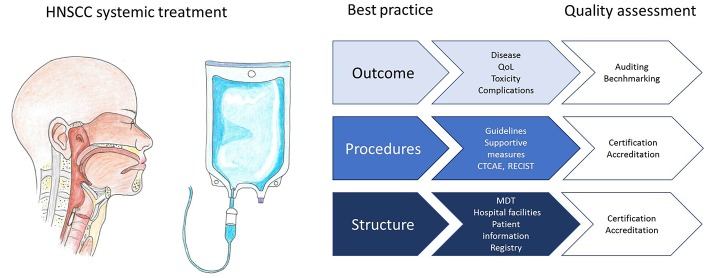
Infographic representing best practice structural requirements, procedures, and outcome evaluation for systemic treatment of head and neck squamous cell carcinoma patients, and how quality can be assessed.

Several accreditation or certification programs have been launched with the aim to improve the quality of care for cancer patients. An example is ASCO's Quality Oncology Practice Initiative (QOPI)^1^. Next to a core module and a symptom/toxicity module, tumor specific modules have been developed, although not yet for head and neck cancer. To illustrate, one of the core module measures is that height, weight, and body surface area should be documented prior to curative chemotherapy. The Organization of European Cancer Institutes (OESI) has created an accreditation and designation program for Clinical Cancer Centers and Comprehensive Cancer Centers which is based on peer review^2^. Participation in such initiatives can help centers to identify and improve evidence based quality indicators (Figure 1). Accreditation programs are mainly focused on structural and procedural quality indicators. Monitoring with benchmarking of outcome parameters is a powerful incentive for implementing best practice procedures, but challenging to achieve, for instance because case mix variability has to be taken into account. Increasingly, national registries of real world data are set up and used for monitoring and improving quality of care (81). The Dutch Head and Neck Audit (DHNA) is a registry that was recently launched and covers a broad spectrum of structural, procedural, and outcome parameters. Participation is mandatory for head and neck cancer centers, and the first results show that even in a small country with centralized head and neck cancer care, variation exists in quality indicators (82). Results of individual centers participating in the DHNA will become publicly available in the next years to maximize transparency and to boost initiatives for implementation of best practice procedures. The registration burden of such initiatives will hopefully decrease in the near future with advanced information technology and registration at the source. Potential draw backs of public availability of institutional results include a risk that institutes will primarily accept low risk patients and that insurance companies may choose to cover costs only in the best performing centers.

In summary, best practice in systemic therapy for HNSCC involves participation in an MDT and following guidelines. It requires detailed knowledge and anticipation of side effects of systemic therapy and expertise in management of this patient population. Documentation of patient characteristics, tumor characteristics, treatment details, and clinical and patient reported outcome is essential for monitoring the quality of care. Participation in initiatives for accreditation and registries for benchmarking institutional results can empower initiatives for implementation of best practice procedures.

## Author Contributions

SO wrote the first draft of the manuscript. RH revised it critically for important intellectual content. Both authors approved the submitted version.

### Conflict of Interest Statement

SO received research grants from Pfizer, Novartis and Celldex. RH received research support from Merck, BMS, Genentech, Pfizer, Kura, and Astra Zeneca and was consultant for BMS, Merck, Genentech, Pfizer, astra Zeneca, GSK, Bayer and loxo.

## Footnotes

^1^https://practice.asco.org/quality-improvement/quality-programs/quality-oncology-practice-initiative (accessed on February 10, 2019).

^2^http://www.oeci.eu/Accreditation/Page.aspx?name=BACKGROUND (accessed on February 10, 2019).
